# Comparison of treatment costs for primary localized prostate cancer in Austria and Vienna: an economic analysis

**DOI:** 10.3389/fpubh.2023.1016860

**Published:** 2023-06-01

**Authors:** Matthias Moll, Gregor Goldner

**Affiliations:** Department of Radiation Oncology, Comprehensive Cancer Center, Medical University of Vienna, Vienna, Austria

**Keywords:** prostate cancer, therapy cost, surgery, radiotherapy, Austria, Vienna

## Abstract

**Background:**

Prostate cancer is the most common cancer in men. Several efficient treatments are available for primary prostate cancer, but an economic comparison of these modalities has not been done in Austria.

**Objective and setting:**

The current study provides an economic comparison of radiotherapy and surgery for prostate cancer in Vienna and Austria.

**Methods:**

We analyzed the catalog of medical services of the Austrian Federal Ministry of Social Affairs, Health, Care and Consumer Protection and present the treatment costs for the public health sector with an LKF-point value and monetary value in 2022.

**Results:**

External beam radiotherapy, especially ultrahypofractionated, is the least costly treatment modality for low-risk prostate cancer, with costs of 2,492 € per treatment. For intermediate-risk prostate cancer, differences between moderate hypofractionation and brachytherapy are small, with costs of 4,638–5,140 €. In a high-risk setting, differences between radical prostatectomy and radiotherapy with androgen deprivation therapy are small (7,087 € vs. 7474.06 €).

**Conclusion:**

From a purely financial point of view, treatment of low- and intermediate-risk prostate cancer in Vienna and Austria should consist of radiotherapy as long as the current catalog of services is up to date. For high-risk prostate cancer, no major difference was found.

## Highlights

- Prostate cancer is the most common cancer in men in central Europe.
- Different treatments, such as radiotherapy and surgery, are effective for tumor control in a primary localized setting.
- In most patients, surgery is more expensive.

## 1. Introduction

Prostate cancer is the most common cancer in men (1, 2), making up almost 20% of all new cancer cases in men (1). With age being one of the main risk factors (2), the number of new cases is expected to increase even further due to the demographics in central Europe.

Several treatment options with excellent oncological results are recommended for primary localized prostate cancer (1–3), including radical prostatectomy, radiotherapy, or, in the case of low-risk prostate cancer, active surveillance. For the purpose of this study, we do not take active surveillance into consideration because it is not a curative treatment on its own and patients may require or desire further primary treatment; 51% of all patients undergoing active surveillance discontinue this type of treatment within 48 months (4). Regarding surgery, patients can be operated on via open access, laparoscopically, or with robot assistance (2). Radiotherapy provides the option of conventional fractionation, hypofractionation, brachytherapy (1, 2), or ultrahypofractionated stereotactic body radiation therapy (SBRT), as well as proton therapy (1). For intermediate- and high-risk prostate cancer, androgen deprivation therapy (ADT) is also recommended, in combination with radiotherapy. However, the German S3-Leitlinie does not recommend proton therapy for primary prostate cancer (2). With our goal of examining the financial treatment conditions in Austria, which has a medical landscape highly influenced by the German guidelines, we excluded proton therapy from our analysis. With plenty of treatment options, the final decision is usually left to the patient, as side effects vary (2). However, the economic aspects of treating the most common cancer in men are usually neglected in this decision.

What is the appropriate way to measure costs in health care? Is it the cost to the provider? Is it the amount of money a patient has to spend for his treatment? Treatment costs according to quality-adjusted life years? Different studies have compared different costs, but there are trends regarding the costs of treatment options. Brachytherapy and SBRT seem to be more cost-efficient than external beam radiotherapy (EBRT) (5, 6), whereas proton therapy and robot-assisted surgery are more expensive than open surgery and conventional EBRT (7). Only one study has covered almost all forms of therapy with the exception of hypofractionated EBRT, showing higher costs of EBRT and brachytherapy compared to surgery, with no major difference between different surgical procedures (8). However, the aforementioned studies almost exclusively cover the US and are, due to differences in the health care systems, barely transferable to Europe. European studies have shown negligible differences between surgery and conventional EBRT (9) and slightly lower lifetime costs for hypofractionated EBRT compared to conventionally fractionated EBRT (10). However, none of the European studies covered open, laparoscopic, and robot-assisted surgery, conventionally fractionated and hypofractionated EBRT, or low dose rate (LDR) or high dose rate (HDR) brachytherapy, especially not for Austria. This study aims to close that gap by evaluating the treatment costs of primary localized prostate cancer in Vienna and Austria.

## 2. Materials and methods

The Austrian public health system is an inpatient or outpatient hospital setting for patients without any private insurance that is financed by a variety of diagnosis-related groups, the so-called “Leistungsorientierte Krankenanstaltenfinanzierung (LKF).” Each medical service is scored and assigned a certain number of points. Each year, each federal state within Austria decides the worth of an LKF-point. Therefore, costs for the same treatment differ by federal state and year. We wanted to evaluate the amount of money spent by the public health sector for each treatment in 2022, as money spent for a treatment cannot be spent otherwise.

Therefore, we aim to provide an overview about prostate cancer treatment costs. As a research method, we performed the following steps.

First, we analyzed the catalog of services (11), published by the Austrian Federal Ministry of Social Affairs, Health, Care and Consumer Protection for relevant codes regarding EBRT, brachytherapy, and surgery. Data collection took place in June 2022. Second, together with our hospital’s controlling team, we researched the number of points for each service and the estimated value of each point in 2022 (the final value is determined the year after). We included only data relevant to treatment of prostate cancer with surgery or radiotherapy, or additional treatment modalities, as supported by the guidelines, such as gold fiducial markers and ADT. Other modalities were excluded. Third, we calculated the cumulative number of points and the cost in € in Vienna for conventionally fractionated, moderately hypofractionated, and ultrahypofractionated EBRT; brachytherapy and open, laparoscopic, and robot-assisted surgery; and gold fiducial marker implantations, as well as, if necessary according to the German S3-guideline (2), ADT. Costs for medication were extracted from the hospital pharmacy in June 2022.

## 3. Results

### 3.1. EBRT and SBRT

We were able to identify the following relevant codes for EBRT and SBRT (11): ZN131, treatment planning, including CT and MRI scans (12) (868 points); ZN172, intensity-modulated radiotherapy (IMRT, including volumetric modulated arc therapy) using a linear accelerator (151 points for each fraction); ZN180, SBRT (661 points for each fraction); ZN174, tracking/controlling of patient positioning (51 points); ZZ533, clinical controls during treatment (30 points); and JG099, further prostatic operations, such as gold marker fiducial implantation (0 points). In the 2022 version of the catalog of services, gold marker fiducial implantation is not assigned any points. Therefore, we replaced it with HDG10.01 B (2,134 points for patients >74 years old) and HDG10.01 C (1,381 points for patients ≤74 years old) for prostate cancer. As the median age of patients is <75 years (13–15), we decided to use HDG10.01 C for further calculations.

The value of each point in 2022 in Vienna was preliminarily assumed to be 0.5676 € for outpatient treatment in a hospital. If a patient is treated in the university hospital, this value is multiplied by 1.17 (= 0.664092 €). For EBRT, we performed a calculation for primary prostate cancer treatment with 78 Gy in conventional 2 Gy fractionation (2) (i.e., 39 fractions), with moderately hypofractionated treatment in 20 fractions of 3 Gy (i.e., total 60 Gy) according to the CHHiP trial (16), as well as 70 Gy in 28 fractions (i.e., 2.5 Gy per fraction) (17), and a calculation for ultrahypofractionated radiotherapy in seven fractions with 6.1 Gy per fraction three times a week (18) as IMRT and SBRT, as well as five fractions with 7.25 Gy to the planning target volume and 8 Gy to the clinical target volume (19). Ultrahypofractionation is only used for treatment of low-risk prostate cancer. We assumed one clinical control per week, eight for the conventional fractionation, four for moderate hypofractionation, and three for ultrahypofractionation. The cost calculations are presented in Table 1.

**Table 1:** Calculation of average treatment costs for EBRT.

Modality	Conventional fractionation (78 Gy in 39 fractions)	Moderate hypofractionation (60 Gy in 20 fractions)	Moderate hypofractionation (70 Gy in 28 fractions)	Ultrahypofractionation, IMRT (42.7 Gy in seven fractions)	Ultrahypofractionation, SBRT (42.7 Gy in seven fractions)	Ultrahypofractionation, IMRT (36.25/40 Gy in five fractions)	Ultrahypofractionation, SBRT (36.25/40 Gy in five fractions)
Treatment in weeks	8	4	6	3 (3 fractions/week)	3 (3 fractions/week)	2 (3 fractions/week)	2 (3 fractions/week)
Treatment planning (points, A)	868	868	868	868	868	868	868
Points per fraction	151	151	151	151	661	151	661
Total points for all fractions (B)	5,889	3,020	4,228	1,057	4,627	755	3,305
Tracking per fraction	51	51	51	51	51	51	51
Points total /tracking (C)	1,989	1,020	1,428	357	357	255	255
Points per control	30	30	30	30	30	30	30
Points total/control (D)	240	120	180	90	90	60	60
Sum A–D	8,986	5,028	6,704	2,372	5,942	1,938	4,488
Viennese public hospital (€)	5100.45	2853.89	3805.19	1346.35	3372.68	1100.01	2547.39
University hospital Vienna (€)	5967.53	3339.05	4452.07	1575.23	3946.03	1287.01	2980.44

IMRT, intensity-modulated radiotherapy; SBRT, stereotactic body radiotherapy.

Patient education (ZZ532, 41 points) has been excluded to keep the EBRT calculation in line with the brachytherapy and surgery calculations, as it is included in the services for inpatients.

The implantation of gold fiducial markers is performed as an inpatient therapy over 2 days. Therefore, the value of each point in 2022 in Vienna is preliminarily assumed to be 0.8105 € in a public hospital, multiplied by 1.17 for the university hospital (i.e., 0.948285 €). In addition, a daily fee of 13 € is added per day in the hospital. For patients with intermediate-risk prostate cancer, we assumed 6 months of ADT with leuprorelin equaling two applications, once every 3 months (544.70 € for 3 months; ZZ533, worth 30 points), as well as 1 month of 150 mg bicalutamide daily (104.50 €/month) according to the DART01/05 GICOR trial (20). Prices were taken from our hospital pharmacy. For high-risk prostate cancer, we assumed 1 month of 150 mg bicalutamide and 18 months of leuprorelin, with 18 months of ADT being equally effective (21) compared to 36 months. The cost calculations for gold fiducial markers and ADT are presented in Table 2.

**Table 2:** Calculated costs for gold fiducial markers and androgen deprivation therapy (ADT).

Gold fiducial marker		
	Points	1,381
	Daily fee in €	13
	Public hospital (€)	1145.30
	University hospital (€)	1335.58
ADT		
	Intermediate-risk medication (€)	1193.90
	Intermediate-risk application (points)	60
	Intermediate-risk total (€), public hospital	1227.96
	Intermediate-risk total (€), university hospital	1233.75
	High-risk medication (€)	3372.70
	High-risk application (points)	180
	High risk total (€), public hospital	3474.87
	High risk total (€), university hospital	3492.24

The patient was assumed to stay in the hospital for 2 days. ADT, androgen deprivation therapy.

### 3.2. Brachytherapy

For brachytherapy via seeds, we were able to identify the following codes (11):

ZN590, transrectal ultrasound for preplanning (57 points as an outpatient service); JG010, implantation of seeds (LDR-brachytherapy) and inpatient care, including patient education (5,634 points, if patients are released within 3 days); ZN221, HDR-brachytherapy and inpatient care, including patient education (1,909 points per intervention, if patients are released within 4 days). In Vienna, HDR-brachytherapy is usually done in three interventions over 3 weeks (22).

For inpatient services, the monetary value of each point is 0.8105 € in 2022. For the University Hospital Vienna, this value is multiplied by 1.17 (= 0.948285 €). In addition, a fee of 13 € per day is added in 2022. The mean duration in the University Hospital Vienna is 2.1 days for LDR and 2.5 days for HDR according to the controlling department. LDR-brachytherapy is only performed in low-risk or favorable intermediate-risk prostate cancer. In this case, no ADT is applied. The results of the cost calculation are presented in Table 3.

**Table 3:** Calculation of average treatment costs for brachytherapy.

Modality		LDR-BT	HDR-BT
Pre-planning ultrasound, outpatient (points)		57	57
Number of interventions		1	3
Intervention and care, inpatient (points)		5,634	1,909
Daily fee (€)		13	13
Costs in a Viennese public hospital (€), assuming average days in the hospital		4637.71	4713.09
Costs in the university hospital Vienna (€), assuming average days in the hospital		5419.49	5507.68

The patient was assumed to stay in the hospital for 3 days in both treatment arms. LDR, low dose rate; HDR, high dose rate; and BT, brachytherapy.

### 3.3. Surgery

We were able to identify the following relevant codes for surgery (11): JG050, open radical prostatectomy (RP) and inpatient care, including patient education (8,744 points, if patients are released between 3 and 15 days); JG060, laparoscopic RP and inpatient care, including patient education (8,744 points, if patients are released between 3 and 15 days); JG070, open RP with lymphadenectomy and inpatient care, including patient education (8,744 points, if patients are released between 3 and 15 days); JG080, laparoscopic RP with lymphadenectomy and inpatient care, including patient education (8,744 points, if patients are released between 3 and 15 days).

For robot-assisted RP, no proper code has been assigned in 2022. Therefore, it is summarized within JG060 and JG080. Concerning the calculation of costs, the same assumptions made for brachytherapy apply here. Mean hospital time for all types of surgery is 9.8 days in the University Hospital Vienna according to our controlling department. The results of the cost calculations are presented in Table 4.

**Table 4:** Calculation of average treatment costs for surgery.

Modality	Any type of RP
Surgery and inpatient care (points)	8,744
Daily fee (€)	13
Costs in a Viennese public hospital (€), assuming 9.8 days in the hospital	7087.01
Costs in the University Hospital Vienna (€), assuming 9.8 days in the hospital	8291.80

RP, radical prostatectomy.

**Figure 1:**
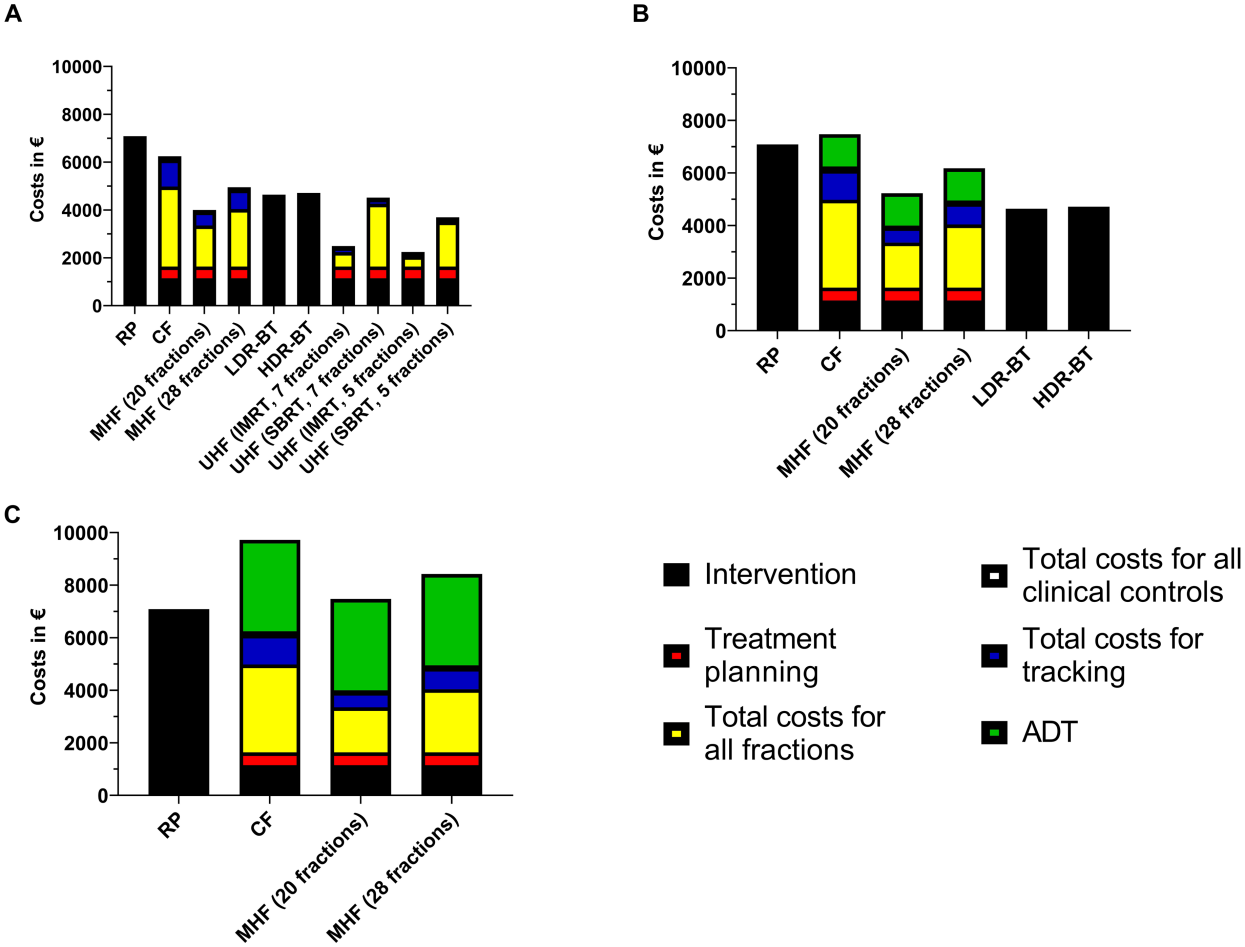
Therapy costs for treatment of prostate cancer in public hospitals. **(A)** low-risk, **(B)** intermediate-risk, **(C)** high-risk. RP: radical prostatectomy, CF: conventional fractionation, MHF: moderate hypofractionation, LDR-BT:low dose-rate brachytherapy, HDR-BT: high dose-rate brachytherapy, UHF: ultrahypofractionation, IMRT: intensity-modulated radiation therapy, SBRT: stereotactic body radiation therapy, ADT: androgen deprivation therapy.

**Figure 2:**
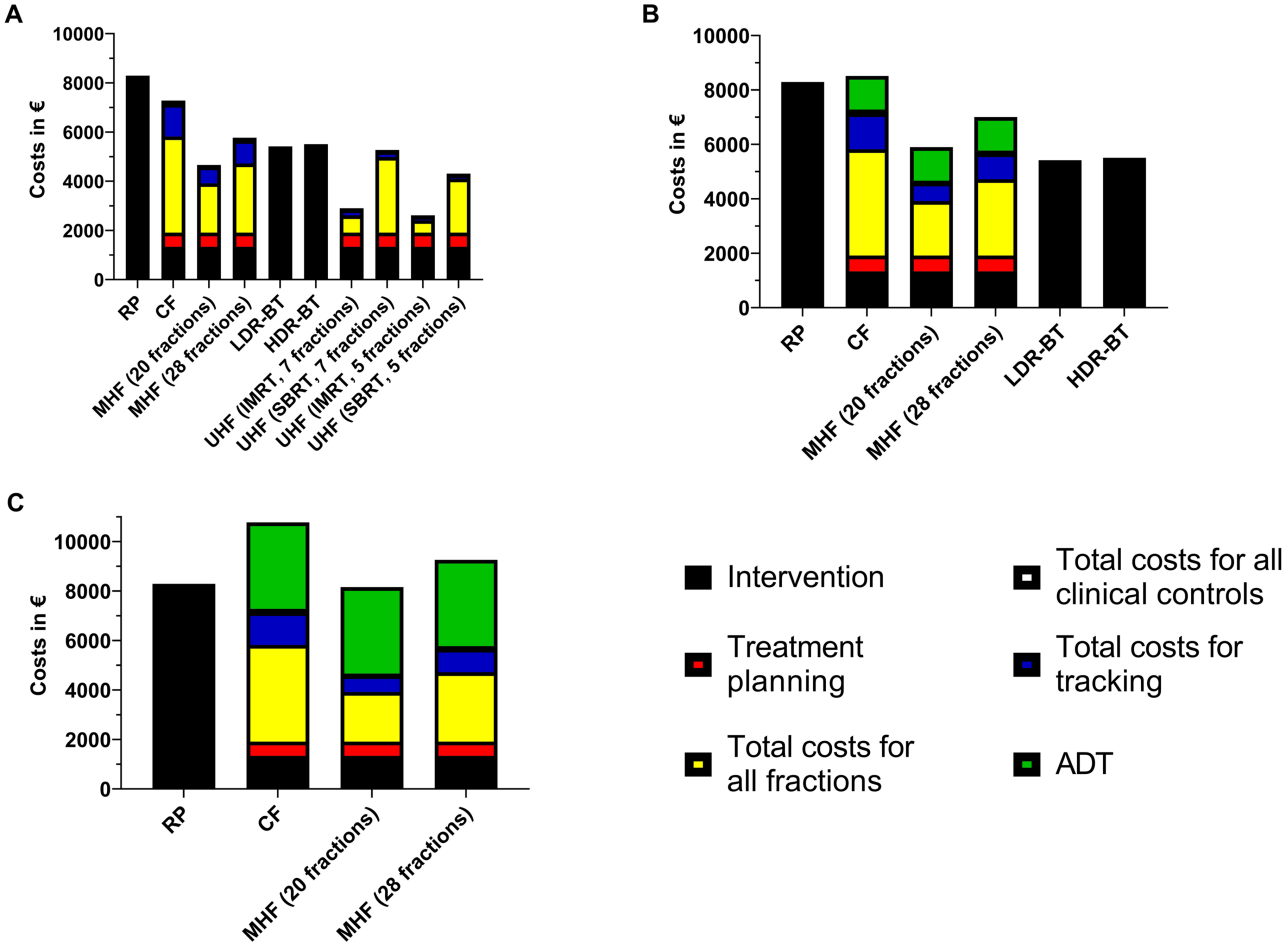
Therapy costs for treatment of prostate cancer in the university hospital. **(A)** low-risk, **(B)** intermediate-risk, **(C)** high-risk. RP: radical prostatectomy, CF: conventional fractionation, MHF: moderate hypofractionation, LDR-BT:low dose-rate brachytherapy, HDR-BT: high dose-rate brachytherapy, UHF: ultrahypofractionation, IMRT: intensity-modulated radiation therapy, SBRT: stereotactic body radiation therapy, ADT: androgen deprivation therapy.

For clarity, all costs are summarized Figure 1 for public hospitals and Figure 2 for the university hospital. We assumed gold fiducial marker implantation for all patients treated with radiotherapy other than brachytherapy and ADT.

## 4. Discussion

Several national guidelines recommend active surveillance, surgery, or radiotherapy for the treatment of prostate cancer (1–3), with comparable oncological results. More than half of all patients discontinue active surveillance (4) and, because it is not a directly curative treatment, was excluded from our analysis.

Looking at our data from a purely financial point of view, radiotherapy, especially when applied with moderate hypofractionation or ultrahypofractionation as IMRT, is the least costly treatment modality for low-risk prostate cancer. Regarding brachytherapy, no relevant differences between LDR and HDR were observed. However, with oncological parity, patient preference should also be taken into consideration. Advantages of surgery and brachytherapy are the one-time intervention, whereas EBRT, even in moderately hypofractionated schedules, takes place over 3–4 weeks, though one-time stereotactic treatments are being evaluated (23). Regarding side effects, EBRT leads to more gastrointestinal side effects but less urinary incontinence and erectile dysfunction within the first 5 years compared to surgery (24), whereas brachytherapy leads to more genitourinary, but less gastrointestinal, toxicity compared to EBRT (13). These facts have to be evaluated, as treatment costs for all modalities in low- and intermediate-risk prostate cancer are well below 10,000 € per treatment, even at the University Hospital Vienna, and side effects may persist for up to at least 5 years (24). Regarding high-risk prostate cancer, radio-oncological treatment and surgery are similarly expensive, mostly due to ADT lasting more than 18 months, accounting for approximately 45% of the overall treatment costs in patients treated with moderate hypofractionation, if delivered in 20 fractions. Comparing both treatment modalities, there are two other important points to consider. On the one hand, there is no randomized controlled trial comparing both treatment modalities, using modern techniques and dose concepts. On the other hand, adjuvant radiotherapy after surgery is recommended in many high-risk prostate cancer patients after surgery (2), such as positive resection margins and/or locally advanced prostate cancer, potentially skewing our results in favor of surgery, as adjuvant radiotherapy is not considered in our treatment costs. New results (25), showing the benefit of ADT in a salvage setting, might also lead to routine ADT use in an adjuvant setting in the future, potentially increasing the difference even further.

The limitations of our study are the transient nature of our results, as potential changes in reimbursement may lead to different results and conclusions in the future. In addition, when looking at monetary values, we only provide data for Vienna. However, proportions stay the same for other federal states, as only the assigned monetary value for each LKF-point changes. Furthermore, though all costs are considered to fully cover the expenses for a treatment according to our controlling department, the profit margins may differ, potentially skewing our results. On top of that, the actually used medication for ADT might differ. On the other hand, looking at the price insurance has to pay provides a broader perspective, as money spent for a specific treatment cannot be spent elsewhere. Besides, costs of treating treatment toxicities are not touched by our study, but might well be a relevant part when considering “overall” treatment cost of a modality.

## 5. Conclusion

From a purely financial perspective, low- and intermediate-risk prostate cancer treatment in Vienna and Austria should consist of radiotherapy as long as the current catalog of services is up to date. A clear economic recommendation cannot be made for high-risk prostate cancer.

## Data availability statement

The raw data supporting the conclusions of this article will be made available by the authors, without undue reservation.

## Author contributions

MM: designing the study, acquisition, analysis, and interpretation of the data, and drafting the manuscript. GG: designing the study and revising the manuscript. All authors contributed to the article and approved the submitted version.

## Conflict of interest

MM has received honoraria from Sanofi-Aventis, unrelated to this submission.

The remaining author declares that the research was conducted in the absence of any commercial or financial relationships that could be construed as a potential conflict of interest.

## Publisher’s note

All claims expressed in this article are solely those of the authors and do not necessarily represent those of their affiliated organizations, or those of the publisher, the editors and the reviewers. Any product that may be evaluated in this article, or claim that may be made by its manufacturer, is not guaranteed or endorsed by the publisher.
