# Supplementary material for: Comparison of treatment costs for primary localized prostate cancer in Austria and Vienna: an economic analysis
Source: Front Public Health. 2023 Jun 1;11:1016860. doi: 10.3389/fpubh.2023.1016860 (PMC10267377; doi:10.3389/fpubh.2023.1016860)
Supplement: Supplementary file 1 [file Table_1.docx]

Supplement: Summary of treatment costs for prostate cancer (Table)

|  | LR |  | IR |  | HR |  |
| --- | --- | --- | --- | --- | --- | --- |
|  | **PH (€)** | **UH (€)** | **PH (€)** | **UH (€)** | **PH (€)** | **UH (€)** |
| Any RP | 7087.01 | 8291.80 | 7087.01 | 8291.80 | 7087.01 | 8291.80 |
| CF | 6245.75 | 7303.11 | 7473.71 | 8536.86 | 9720.62 | 10795.35 |
| MHF (20 fractions) | 3999.19 | 4674.64 | 5227.15 | 5908.38 | 7474.06 | 8166.87 |
| MHF (28 fractions) | 4950.49 | 5787.65 | 6178.45 | 7021.40 | 8425.36 | 9279.89 |
| UHF (IMRT, 7 fractions) | 2491.65 | 2910.81 | - | - | - | - |
| UHF (SBRT, 7 fractions) | 4517.98 | 5281.62 | - | - | - | - |
| UHF (IMRT, 5 fractions) | 2245.31 | 2622.59 | - | - | - | - |
| UHF (SBRT, 5 fractions) | 3692.69 | 4316.03 | - | - | - | - |
| LDR-BT | 4637.71 | 5419.49 | 4637.71 | 5419.49 | - | - |
| HDR-BT | 4713.09 | 5507.68 | 4713.09 | 5507.68 | - | - |

LR = low-risk prostate cancer, IR = intermediate-risk prostate cancer, HR = high-risk prostate cancer, PH = public hospital, UH = university hospital, RP = radical prostatectomy, CF = conventionally fractionated EBRT, MHF = moderately hypofractionated EBRT, UHF = ultrahypofractionated radiotherapy, IMRT = intensity-modulated radiotherapy, SBRT = stereotactic body radiotherapy, LDR = low dose rate, HDR = high dose rate, BT = brachytherapy
